# Metallurgical investigation on fourth century BCE silver jewellery of two hoards from Samaria

**DOI:** 10.1038/srep40659

**Published:** 2017-01-18

**Authors:** D. Ashkenazi, H. Gitler, A. Stern, O. Tal

**Affiliations:** 1 School of Mechanical Engineering, Tel Aviv University, Ramat Aviv 6997801, Israel; 2 Israel Museum, Derech Rupin 11, Jerusalem 9171002, Israel; 3 Department of Materials Engineering, Ben-Gurion University of the Negev, Beer Sheva 8410501, Israel; 4 Department of Archaeology and Ancient Near Eastern Cultures, Tel Aviv University, Ramat Aviv, 6997801, Israel

## Abstract

A fourth century BCE silver jewellery collection, which is part of two hoards of Samarian coins (the Samaria and Nablus Hoards), was studied by non-destructive analyses. The collection, which consists of pendants, rings, beads and earrings, had been examined by visual testing, multi-focal microscopy and SEM-EDS analysis. In order to enhance our knowledge of past technologies of silver jewellery production, we developed a metallurgical methodology based on the chemical composition of the joints and bulk. The results show that all artefacts are made of silver containing a small percentage of copper. Higher copper concentrations were measured in the joining regions. Our research indicates that the manufacturing of the jewellery from both hoards involved similar techniques, including casting, cutting, hammering, bending, granulating and joining methods, indicating that the artefacts were made by trained silversmiths. Although the burial date of the Samaria Hoard – 352 BCE – is some 21 years earlier than that of the Nablus Hoard – *circa* 331 BCE, a noted continuity in the local production technology is apparent in the analysed items. This information provides better understanding of the technological abilities in the late Persian-period province of Samaria and bears implications on the local silver coins produced in the region.

The items examined are part of the silver jewellery assemblage from the Samaria Hoard (Fig. 1), whose burial date was 352 BCE, and from the Nablus Hoard, whose burial date is *circa* 331 BCE. The items from the Samaria Hoard include a ring, two pendants, a bead and a jewellery fragment (Fig. 2); those from the Nablus Hoard include a ring, four pendants, a few silver beads and two earrings (Fig. 3).

After the Six Day War in 1967 and the occupation by Israel of what is now known as the West Bank, numerous antiquities appeared on the Jerusalem market as the inhabitants of the newly occupied territories realized that there was a good market for antiquities in Israel. Among these, two fourth century BCE coin hoards with jewellery appeared on the market in about 1968. One of them, known as the Samaria Hoard allegedly included 334 coins and several pieces of jewellery and was found in a pottery container. The vessel, along with 34 coins and the jewellery, are part of the Israel Museum collection (Inv. Nos 93.016.14531–14569). Information about this hoard was published by Meshorer and Qedar (1991) and also by Meadows and Wartenberg (2002) (=*CH* 9.413 Samaria, before 1990) and Elayi and Elayi 1993^1^,^2^,^3^. The former^1^ mentioned another hoard in their publication, the Nablus Hoard (*IGCH* 1504 =*CH* 9.440, Nablus, 1968. See also Elayi - Elayi 1993, pp. 231–239), which allegedly also included some jewellery but which was sold in several lots in the Jerusalem antiquities market without a container. Based on a short description by Arnold Spaer and Silvia Hurter^2^,^4^, this hoard contained 965 coins plus jewellery. There have been suggestions that the Nablus Hoard was actually part of the Samaria Hoard and that both these hoards originated with the finds from Wadi ed-Daliyeh in the Jordan Valley that have been attributed to refugees from the persecutions of Alexander the Great after he conquered Samaria. Spaer, who owned about half of the Nablus Hoard, mentioned in a note he published in 2009 that none of these suggestions was correct and that the location of the Samaria Hoard, although the hoard was found as a unit, had not been named^5^. The Nablus Hoard, on the other hand – based on the information Spaer received from the dealers connected with the find – was said to have been found in the village of Jinsafut along the Qalqilyah – Nablus road, whilst according to other information, it was found near Kutsra, north of Shiloh. Spaer’s assumption is corroborated by the fact that there are no die-links between the two hoards with the exception of isolated examples. We can thus logically assume that these are two separate finds^6^.

The dating of the burial of the two hoards found in Samaria is mainly based on the dating of the Sidonian and Tyrian issues found in them since the inner chronology of these coinages is well attested^7^,^8^. The accepted date for the burial of the Samaria Hoard was 355 BCE based on the latest dated Sidonian issue in this hoard – a quarter *sheqel* of ‘Abd‘aštart I dated to Year 14 BCE^3^. However, according to the new chronology of the Sidonian kings, Year 14 of ‘Abd‘aštart I (365–352 BCE) falls in 352 BCE^9^,^10^. Thus, the burial date of the Samaria hoard should be set to after this date. Moreover, a post 352 BCE date for the burial of the Samaria Hoard is evident from the fact that the vast majority of the local Athenian-styled Palestinian ‘obols’ and ‘hemiobols’ in this hoard imitate Athenian *pi-style* Owls which are dated to after 353 (to about 295 BCE)^11^,^12^. On the other hand, the latest dated issue in the Nablus 1968 Hoard is a previously unpublished Sidonian 1/16 of a *sheqel* of Mazday (353–333 BCE) dated to Year 21 (=333 BCE)^7^. This issue determines a *terminus post quem* for the burial of the hoard. It is tempting to connect the burial date of this hoard to the political reality of 331 BCE. The Aramaic legal and administrative papyrus documents from Wadi ed-Daliyeh constitute a representative group from the end of the Persian period in Palestine that is indirectly related to the event described by Rufus (History of Alexander IV, viii, 9–11) – the killing of Andromachos, Alexander’s appointed governor – which resulted in the flight of the Samaritan elite to hiding complexes in the hideout caves of Wadi ed-Daliyeh with their most precious and portable belongings^13^.

Silver is one of the first metals used by early civilizations; therefore, silver objects such as the present silver jewellery assemblage from the Samaria and Nablus Hoards, represent the material cultural heritage of certain populations in certain periods^14^. Hence, numerous studies of ancient silver artefacts exist in the literature, investigating their chemical composition^15^,^16^,^17^,^18^,^19^,^20^,^21^, microstructure^15^,^17^,^20^,^22^,^23^, manufacturing processes^15^,^24^, provenance^16^,^17^, embrittlement and fracture^22^,^23^, condition of preservation, corrosion processes and corrosion products^14^,^25^,^26^,^27^,^28^,^29^ and their state of conservation^25^,^29^. These studies normally combine non-destructive testing (NDT) and destructive testing methods, including: metallography, light and scanning electron microscope (SEM) examination including energy-dispersive spectroscopy (EDS) analysis, focused ion beam (FIB) microscopy and micromachining, particle-induced X-ray emission (PIXE) analysis, X-ray fluorescence (XRF) analysis, inductively-coupled plasma (ICP) analysis, X-ray diffraction (XRD), neutron tomography, X-ray photoelectron spectroscopy analysis (XPS), and Raman spectroscopy analysis^15^,^16^,^17^,^18^,^19^,^20^,^21^,^22^,^23^,^25^,^26^,^27^,^28^,^29^,^30^,^31^,^32^. Yet, because of the rareness of such ancient artefacts, from the archaeological perspective, the use of NDT methods is always preferred^20^,^21^,^30^.

Silver was commonly used in antiquity to manufacture objects such as jewellery, ornaments and coins^33^, where there was need for sophisticated technological skills, such as casting, plastic forming, cycle of cold working (hammering) and heating (annealing), granulation and joining techniques^15^,^24^,^34^.

Copper is considered a main alloying element commonly found in silver jewellery; and the presence of more than 2.6 wt% Cu suggests that the copper was intentionally added to the silver^35^,^36^,^37^. Copper, widely used as a melting-point depressant, diffuses easily in silver, and is therefore frequently applied as a joining material (filler metal) of silver components^24^,^34^.

Metal granulation was used to produce and join rounded metal particles called granules (or globules)^38^,^39^. The tiny granules are formed when molten-metal droplets impact a solid substrate by fusing the end of a silver wire^39^. Granules may also be formed when a silver wire is inserted into a crucible and suspended in powdered charcoal until it melts. The most complicated expertise associated with the granulation process is how to join the granules together and how to join the granules onto a flat or curved sheet of metal^39^.

Metallurgical joins are defined as bonds between metal parts on an atomic level to form a continuous workpiece^34^. Such joins, occasionally with the addition of an intermediate layer, are durable and efficient when the interdiffusion process of atom penetration within the two metal joint components is achieved^34^.

A brazing process is defined as a joining process in which metal parts are joined together by melting a filler metal, with a lower melting point than the joining metal, into the joint without melting the work pieces. In this process a metallurgical joint is created between the filler metal and the surfaces being joined. A brazing process was already used around 3000 BCE to join silver parts by using silver-copper alloys^32^. An interlayer of a near-eutectic Ag-Cu alloy was placed between two pieces of silver, and next the Ag-Cu sandwich was heated to the ‘joining’ temperature (above T_E_); through subsequent cooling, the liquid alloy solidifies, creating a metallurgical joint^40^,^41^.

The fusion of two metals in the area of contact when the temperature is below the fusion points of each metal is defined as contact melting (CM)^42^. The CM phenomenon, is rather common and has important manufacturing implications for ancient jewellery production^42^,^43^. In a previous work on binary systems and CM, the following conclusions were obtained^42^: (1) during the heat-up stage, at temperatures near and below the eutectic temperature, the solid-solid interface forms a diffusion zone (DZ) with a typical width of a few microns; (2) for the temperature range of 10–20 *°*C above the eutectic point (under isothermal conditions), the formation of the liquid eutectic layer at the joint interface is observed, which is preceded by the formation of the DZ. During the cooling, the solid/liquid interface shifts toward the joint line and, as soon as the two solid/liquid interfaces meet, the solidification process ends, resulting in the creation of a metallurgical bond^42^. CM is applicable to eutectic alloy systems, where a significant reduction of the liquidus temperature occurs during alloying^42^. For a binary A-B eutectic system, an interlayer of material B is placed between two pieces of material A. The sandwich is then heated to the ‘joining’ temperature (above the eutectic point, T_E_). As a result of the contact between A and B atoms, the interlayer melts and the molten region further widens by dissolving A atoms. Thus, further liquid is created, homogenizing by diffusion of B atoms into the neighboring solid and causing a reversal of the widening behaviour^40^. Through subsequent cooling, the liquid undercools below T_E_ and solidifies, creating a metallurgical joint^34^,^40^. The binary phase diagram of Ag-Cu^15^,^32^,^40^ (Fig. 4) properly describes the eutectic system being examined in the current study. In antiquity it was possible to produce silver-silver joints by adding a copper compound plus organic slurry-glue to the joining zone, followed by the heat reduction to a copper interlayer, forming dedicated joints, depending on the process conditions^43^. Different copper compounds could have been used to create metallic copper layers on the silver surface: (1) powdered Malachite mineral, Cu_2_(OH)_2_CO_3_, was used according to Pliny the elder^32^,^39^,^44^,^45^,^46^; (2) cupric oxide (CuO) black powder, produced by grinding the oxide formed on the copper surface after annealing^46^,^47^,^48^; (3) copper chloride dehydrate, CuCl_2_(H_2_O)_2_, obtained by annealing a copper sheet painted with salt^48^,^49^; and (4) Chalcanthite mineral (CuSO_4_·5H_2_O)^48^,^50^.

When a silver workpiece is heated with a reducing flame (with low oxygen), the copper is released from its compound and the organic slurry/glue will disappear. As the eutectic temperature is reached, the small amount of copper at each point of contact (Ag-Cu-Ag interface) will form a liquid eutectic layer creating a local joint after the cooling and solidification.

It is rare and challenging to find literature on the joining technologies and joint composition of such ancient silver objects^24^,^32^. Moreover, none of these studies has developed a metallurgical methodology comparing the chemical composition of the joints of such silver objects to their bulk composition based on NDT methods in order to understand their manufacturing processes. The present research attempts to fill this gap.

Although only 66 Samarian silver coins were examined by XRF method more than a decade ago^51^, Achaemenid-period jewellery found in Palestine have not yet been chemically analysed. Furthermore, other contemporaneous fourth century BCE indigenous silver coins of the Achaemenid-period provinces of Palestine were subject to traditional archaeo-metallurgical analytical approaches, especially XRF and ICP with atomic emission spectrometry (AES)^35^.

In this article, we present for the first time a NDT metallurgical methodology for analysing ancient silver jewellery from two known hoards, based on their manufacturing techniques and by determining the chemical composition of the Ag-Ag joints. This information provides better understanding of Persian-period technological abilities in the southern Levant.

## Results

Visual testing (VT), stereo and multi-focal light microscopy (LM) and SEM-EDS analysis revealed that related production techniques were involved in the manufacturing processes of the jewellery from both hoards, including casting, cutting the metal foil into the desired shape, decorating the object by applying plastic deformation (hammering and notching), twisting a thin strip into a wire, as well as producing small granules and then joining them together, or joining granules to the jewellery.

**Figure 2:**
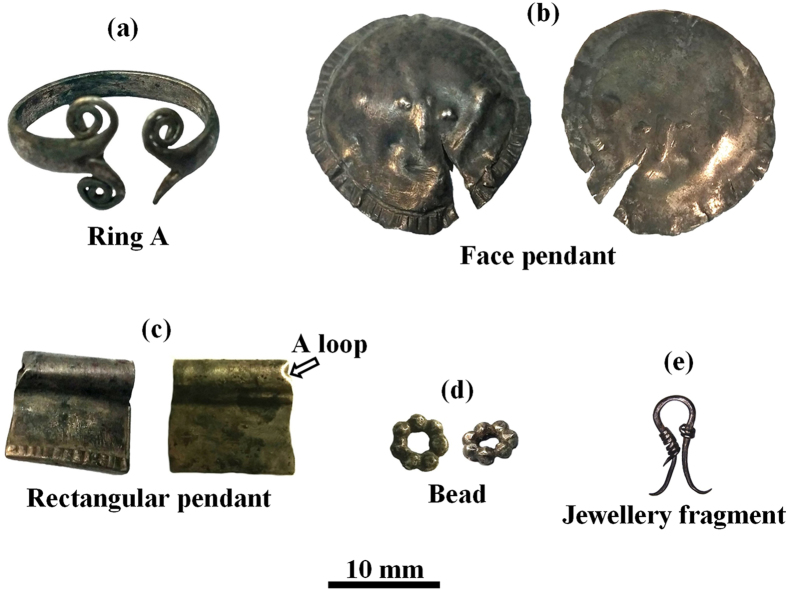
Selected silver jewellery from the Samaria Hoard: (**a**) spiral ring A; (**b**) face pendant, front and back (left and right images, respectively); (**c**) rectangular pendant, front and back (left and right images, respectively); (**d**) single bead made of small globules (left: upper view, right: isometric image); and (**e**) jewellery fragment. Photographer: D. Ashkenazi and P. Shrago.

The spiral silver ring A from the Samaria Hoard (Fig. 2a), with total weight of 2.17 gr, was found in a good state of preservation. No joints were observed. The ring was produced from a cast bar that was flattened by hammering. Then both ends of the ring were split into two halves, cutting a 12 mm lengthwise groove. Three edges were then bent into spirals (Fig. 5a and b). The fourth edge may have been shortened and bent into a branch shape, or its spiral may have been broken off and lost. Then the bar was plastically deformed and bent into a final ring.

The silver face pendant from the Samaria Hoard (Fig. 2b), with total weight of 1.09 gr and average thickness of 0.42 mm, was relatively well-preserved. However, it was found with a 10.5 mm long cut running diagonally (Fig. 2b). No evidence for top loop and joints was observed. Therefore, it seems that the manufacturing process was not completed and a lavalier was not attached to the item. The face pendant was produced from a cast ingot, flattened by hammering, that was cut into a round shaped sheet. Next the back of the object was hammered to produce a shallow-relief rough face, and its front was hammered to form the mouth and the round frame ornament, decorated with geometrical patterns (Fig. 5c and d).

The silver rectangular pendant from the Samaria Hoard (Fig. 2c), with a total weight of 0.3 gr, was found in a very good state of preservation. The average thickness of the pendant near its loop was 0.16 mm and the average thickness of the lower end was 0.62 mm. It was produced from a cast ingot, flattened by hammering to create a rectangular sheet. Next the sheet was folded in half, creating a top cylindrical loop (with external diameter of 3.2 mm). The 2 mm lower part of the object was then bent and folded again and a mechanical joint was created. Next, the rectangular pendant was decorated with straight strips (Fig. 5e) made with a sharp-pointed hand tool.

The silver bead from the Samaria Hoard (Fig. 2d), with total weight of 0.17 gr, was found in a very good state of preservation. It was made of seven globules, each with an average diameter of 1.4 mm, which were joined together (Fig. 5f). Narrow gap joints were observed between granules.

The silver jewellery fragment from the Samaria Hoard (Fig. 2e), with total weight of 0.11 gr, was found in a good state of preservation. It was made of a 700 μm diameter wire, which was plastically deformed into an omega-shaped part with sharp tips, and then it was decorated by 400 μm wire wrapping produced by twisting a strip cut from a very thin sheet. This wire was wrapped around the left and right flanks of the object, creating a decoration of two coils (Fig. 5h and i, respectively). Next the wire was joined to the bar. Wide gap joints can be observed between the spiral wires and the main wire.

**Figure 3:**
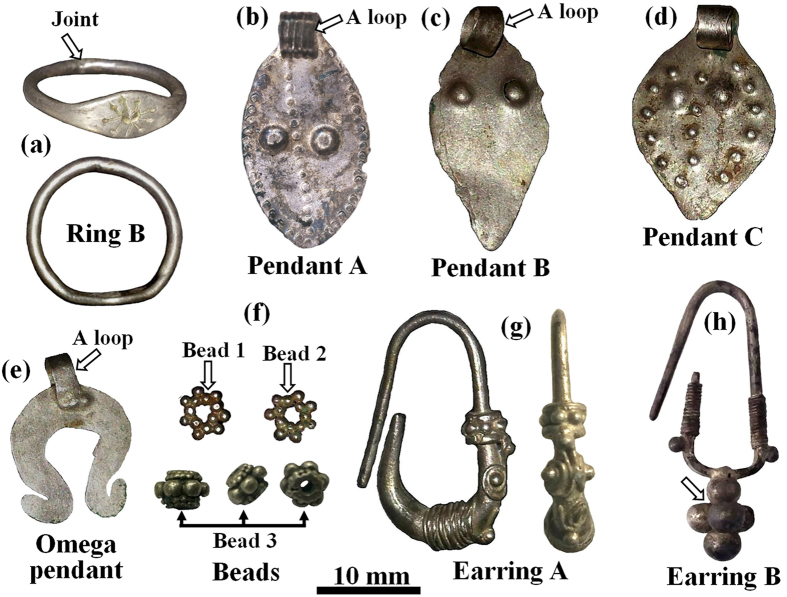
Selected silver jewellery from the Nablus Hoard: (**a**) decorated ring B (front and top views); (**b**) leaf-shaped pendant A (front); (**c**) leaf-shaped pendant B; (**d**) leaf-shaped pendant C; (**e**) omega pendant (front); (**f**) beads made of small globules (beads 1 and 2, top view; and bead 3, front, isometric and top views); (**g**) decorated earring A (left: side A, right: front of earring); and (**h**) decorated earring B (side A). Photographer: D. Ashkenazi and P. Shrago.

Ring B from the Nablus Hoard (Fig. 3a), with total weight of 2.55 gr, was found in a good state of preservation. It was made from a cast silver bar, which had been flattened in its middle section by hammering. Then the flat area was decorated by a flower-shaped engraving; finally, the bar was bent and its ends were joined to produce a ring. A narrow gap joint can be observed at the back of the ring.

The A-B-C leaf-shaped pendants from the Nablus Hoard (Fig. 3b–d), with total weights of 0.70, 0.28 and 0.45 grams respectively and an average thickness of 0.15–0.21 mm, were found well preserved. They were made of cast silver ingots, flattened by hammering to produce a thin sheet. Next, each foil was cut into the desired leaf shape with a petiole at its upper part. No joints were observed. The back of each object was hammered to produce the decoration marks and the leaf stem was bent to create the loop of the lavalier.

The omega pendant from the Nablus Hoard (Fig. 3e), with a total weight of 0.55 gr and an average thickness of 0.45 mm, was found in a good state of preservation. It was produced from cast ingot-shaped metal that had been flattened by hammering to produce a thin sheet; next the silver foil was cut into an omega shape. Then a 2.3 mm strip was bent and joined to the pendant to create the lavalier (Fig. 6). The loop at the upper part was prepared by simultaneously brazing a U-shaped silver piece to both sides of the pendant. Wide gap joints can be observed in both front and back sides of the pendant (Fig. 6b and c).

The beads from the Nablus Hoard (Fig. 3f, beads 1 and 2), with an average weight of 0.15 gr, were made of small granules which were joined together simultaneously to form a bead. Beads 1 and 2 (Fig. 7a and b respectively) are composed of 14 granules with an average diameter of 800 μm; and bead 3 (Fig. 7c and d) was made of large (1.5–1.8 mm) and small (500 μm) globules. Narrow gap joints were detected between the granules. The exceptional bead 3 (Fig. 3f) was manufactured from two beads made of tiny granules and one bead made of a larger granule that had been joined together.

Earring A from the Nablus Hoard (Figs 3g and 8a–d), with total weight of 1.89 gr, was found in a good state of preservation. In order to manufacture the earring, first a silver alloy was cast, and then a rod (with a maximal diameter of 3.2 mm) was hammered to produce the initial earring hoop. Next the rod was decorated by wrapping wire (made of a twisted 100 μm thick silver sheet) around the rod and by adding small granules (with an average diameter of 800 μm), including a bead made of granules (Fig. 8d). At one location (surrounded by a silver wire) a green corroded narrow gap area was detected where a globule had probably once been located (now missing) as typical to copper oxide (Fig. 8b).

Earring B from the same hoard (Figs 3h and 8e–h), with a total weight of 1.49 gr, was found relatively well-preserved. It was made of a cast silver bar whose lower part had been hammered to form a square cross-section; the bar was then bent to the desired shape. Next, the earring hoop was decorated by wrapping wire on both arms and by joining small globules, with an average diameter of 1.8 mm, to the coil end (Fig. 8f–h). The lower part of this earring contains five large decorative globules, each of about 5 mm in diameter. The earring was assembled in two parts: the multi-joint earring with narrow gap joints and the large globules. The last manufacturing step was to join the large upper globule assembly to the square cross-section of earring B’s rod (Fig. 8a).

Although the jewellery from both silver coin hoards are relatively well preserved, EDS analysis of the surface of the objects revealed the presence of oxide, some corrosion products, and soil remains (Tables 1 and 2). For example, examination of ring A from the Samaria Hoard (Fig. 5a–b) revealed the presence of the elements Ag, Cu, O, Si, S and Ca (Table 1) and examination of the ring B from the Nablus Hoard (Fig. 3) revealed the presence of the elements Ag, Cu, O, Si and Cl (Table 2). The presence of about 3.5 wt% Pb was detected at the back of ring B (Table 2); such a lead concentration is most likely related to the extraction process of the silver from lead by cupellation refining process^16^,^36^.

EDS could not be used as a quantitative tool for chemical analysis without careful consideration of the error associated with this technique. Therefore, before measuring the composition of the silver jewellery from both hoards, 80 Samarian silver coins from the Samaria and Nablus Hoards as well as other coins from the Israel Museum collection were chosen to be examined by EDS analysis. These coins were selected from over 600 Samarian coins stored at the Israel Museum based on visual testing in order to avoid examining coins which are corroded or radically cleaned. After having selected a group, we further eliminated additional coins based on our SEM observations. From the 80 selected coins, 2–4 areas of each coin were meticulously selected to be tested according to their better preserved surface, in order to estimate the error associated with the method. The average measured composition of all 80 silver alloyed coins (after omitting the peaks of oxides and soil elements) was 95.9 ± 2.5 wt% Ag and 4.1 ± 2.4 wt% Cu.

EDS chemical analysis of the jewellery from both hoards showed that all items were made of silver alloys containing a small wt% of Cu and above (Table 1 and 2). For instance, the silver alloy of the face pendant from the Samaria Hoard (Fig. 5d) contained 4.6–7.3 wt% Cu; the rectangular pendant (Fig. 5e) contained 5.7–6.1 wt% Cu (Table 1); and the silver leaf-shaped pendant C from the Nablus Hoard contained only 1.8 wt% Cu (Table 2). Additionally, the composition of ring B from the Nablus Hoard was examined before and after fine grinding. The surface region contains roughly the same copper composition in the silver alloy (Ag/Cu ratio) as the bulk of ring B. The results revealed that the silver alloy composition at the back of ring B (no grinding) contained 5.3 wt% Cu; and the composition at the back after fine grinding contained a similar amount of copper of 3.9 wt% Cu (Table 2).

It is worth noting that a higher amount of copper was observed in the silver alloy of ring A from the Samaria Hoard, which contained 12.4–25.3 wt% Cu (Table 1); as well as in pendant B from the Nablus Hoard, which contained 13.6–35.7 wt% Cu and in bead 1’s globules which contained 13.2–14.7 wt% Cu (Table 2).

A significantly higher concentration of copper was measured within the joints than in the surrounding parts. For example, EDS examination of the omega pendant front (loop, Fig. 6a) revealed that it was composed of silver with 4.3 wt% Cu. However, the joined area to the loop was made of a silver containing 9.7 wt% Cu (Fig. 6b, Table 2). The back of the same pendant revealed similar results, with 6.9 wt% Cu at the loop and 20.1–29.7 wt% Cu in the joint area (Fig. 6c, Table 2). Examination of the bead from the Samaria Hoard (Fig. 5f) revealed that its silver alloy contained 4.6–5.2 wt% Cu and the joining region between two globules contained 14.4–29.4 wt% Cu (Table 1). Examination of bead 1 from the Nablus Hoard (Fig. 7a) revealed that its joint contained 42.4 wt% Cu (Table 2). Beads 2 and 3 from the Nablus Hoard (Fig. 7b and c, respectively) also revealed a higher concentration of copper within their joints (Table 2). The bar of the jewellery fragment from the Samaria Hoard (Fig. 5g) was made of silver containing 6.5–8.8 wt% Cu, and the coil contained 9.8 wt% Cu; nevertheless, the alloy at the joint contained 38.6 wt% Cu (Fig. 5g–i, Table 1). In earring A from the Nablus Hoard, the silver alloy at the bar contained 3.7–3.8 wt% Cu, and the coil contained 2.2 wt% Cu; however, the joint between the globule and the coil contained 13.7 wt% Cu (Fig. 8b). Earring B from the Nablus Hoard (Fig. 8f–h) revealed similar results (Table 2).

The Ag/Cu ratio, between the near-surface and the bulk of the jewellery also remained relatively constant at the joints. For example, the silver alloy composition of the back of ring B (joint, before grinding) contained 8.8 wt% Cu; and the composition of the back (joint, after grinding) was 9.5 wt% Cu.

## Discussion

A collection of fourth century BCE silver jewellery from the only two known hoards of Samarian coins and jewellery, the Samaria and Nablus Hoards, was studied by metallurgical NDT analysis. Since Ag_2_O is a stable oxide, silver generally has excellent corrosion resistance^33^ and indeed the jewellery under study was found to be in a good state of preservation according to the VT and stereo microscope observations. However, the thickness of the oxide layer may affect the EDS measurements, especially when the oxide layer is thick, which is frequently seen on Ag-Cu artefacts^52^. The EDS analysis of the jewellery surface revealed the presence of the elements O, Si, Cl, S, Mg, Fe and Ca (Table 1 and 2). These results were expected since silver is sensitive to chloride and sulphide ions, resulting in the occurrence of silver sulphides (Ag_2_S) and chlorides (AgCl) as main contamination products^28^,^29^,^30^,^33^. Soil element markers such as Si, Mg, Ca, Al, and Fe are also common in archaeological silver artefacts since these elements usually interact with corrosion products of the buried artefacts^29^. Nevertheless, the presence of Cl and S in silver joints often provides a hint as to the type of compound that was used during the joining process^49^,^50^.

EDS is a surface technique; therefore, surface measurements of ancient silver objects may not provide reliable information of the bulk composition, due to tarnish layers, corrosion, oxide layers, conservation treatments, cleaning residues, and silver enrichment of the surface^16^,^18^,^19^,^20^,^21^,^36^,^53^,^54^. Therefore, in order to achieve an optimal (relatively quantitative) SEM-EDS chemical analysis that will represent the bulk of the object, it is essential to understand the limitations of the EDS measurements. Consequently, different parameters should be taken into account during the examination of the object as well as during the interpretation of the results, among them the operation parameters and the calibration of the EDS system, the condition of the examined specimen’s surface, including complex topography and presence of cracks as well as the condition of the preservation of the object^55^. Nevertheless, these limitations do not prevent SEM-EDS from being a functional tool for the study of ancient Ag-Cu alloys. According to these limitations and difficulties associated with EDS tool, it has been suggested by Carl and Young^21^ to combine SEM-EDS measurements to other techniques such as FIB.

From the perspective of archaeology and numismatics, determination of the original alloy composition provides valuable information^20^. Therefore, it is important to determine whether the composition of the surface layer is different than the bulk composition^54^. In Ag-Cu alloys, long period burial in aggressive soil environments may lead to a local selective galvanic corrosion attack of the copper-enriched areas and may lead to immigration of copper atoms from the bulk to the surface of the archaeological silver object, resulting in cuprite (Cu_2_O) on the external surface^15^. The thickness of this oxide layer will affect the external surface measurements, and therefore the quantification of such surface analysis is questionable^19^,^20^. Furthermore, the corrosion layer of ancient silver objects such as coins is not always uniform and may exhibit dissimilar thicknesses at different examined areas of the same artefact^30^. The thicknesses of the oxide formed on top of ancient silver objects usually ranges between 25–250 μm^19^,^31^, depending on the age of the object as well as on the aggressiveness of the long burial environment^31^, but may also be affected by the aggressiveness of the compounds present in the museum atmosphere^28^,^29^. On the other hand, ancient Ag-Cu artefacts often demonstrate silver surface enrichment, which leads to reduction of the copper amount on the external surface^16^,^18^,^19^,^20^,^30^,^54^,^56^.

In the case of the present silver jewellery and coins from both hoards, based on their shiny metal surfaces, the artefacts were most likely cleaned^16^. Nevertheless, the cleaning methods were not recorded and remain unknown. However, even in such circumstances, surface analysis can be used to characterize Ag-Cu artefacts without any further polishing of the surface when the remaining oxide and corrosion layers are thin enough^37^,^52^. The comparison between the Ag/Cu ratio at the surface of ring B (before grinding) and the Ag/Cu ratio at its bulk (after grinding) leads to the conclusion that the Ag/Cu ratio remains relatively constant (Table 2), and therefore the EDS measurements represent the bulk concentration of the objects quite well. ICP-AES and XRF examination of southern Palestinian Persian period silver coins also support this conclusion^35^.

Three joining techniques were used to execute the jewellery belonging to the two hoards: 1. **mechanical joining** by folding straight-edged sheets, e.g., the rectangular pendant; and by two welding techniques based on melting and solidification of filler metals; 2. by the **CM technique**^42^; and 3. by **eutectic brazing**^38^. Intricate pieces, containing many joints (multi-joint jewellery), were most often created by the CM in order to create all the joints in one step and therefore minimize the risk of remelting previous joints and leading to joining failure. The relatively simple items, typically containing one or two joints, were usually executed using a brazing technology. Brazing was accomplished using filler metals made of near-eutectic Ag-Cu alloys. In some of the presently examined jewellery, a near eutectic alloy was added in order to join the parts, whereas in other cases copper compounds were locally added to the interface to create thin copper layers during the joining process (CM).

**Figure 4:**
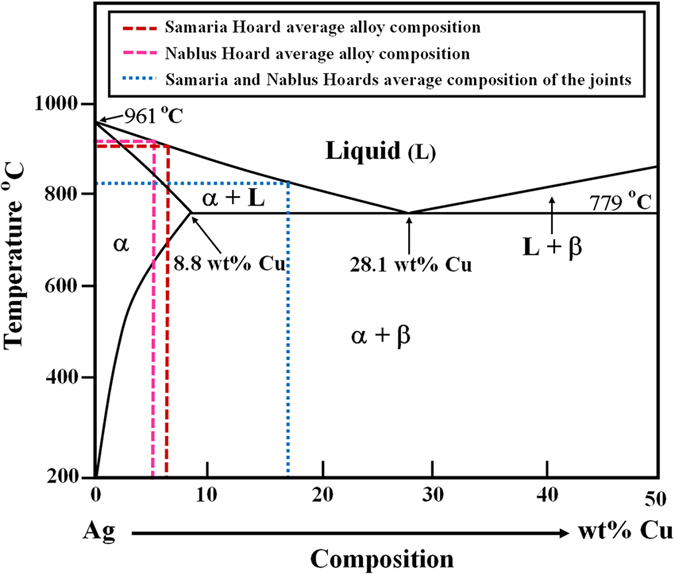
Binary eutectic phase diagram of the Ag-Cu system (based on literature^15^,^32^,^40^). The dash lines are the average general alloy compositions and the average compositions (wt%) of the joining areas, for jewellery from both Samaria and Nablus hoards. The average copper concentration in the jewellery from the Samaria and Nablus Hoards was 6.6 ± 1.6 wt% Cu and 5.1 ± 1.9 wt% Cu, respectively; the average copper concentration in the joints from both hoards was 17.0 ± 10.0 wt% Cu.

**Figure 5:**
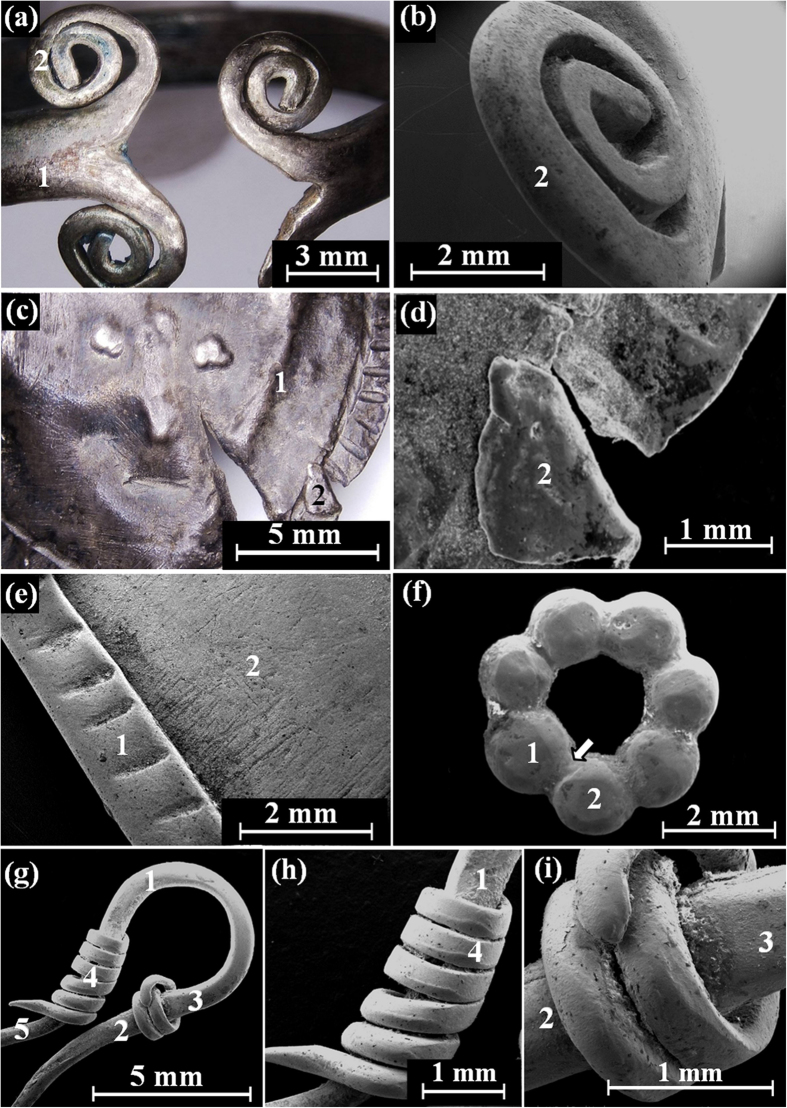
The silver jewellery from the Samaria Hoard: (**a**) the front of ring A (LM); (**b**) one of the spirals of ring A (SEM); (**c**) the face pendant (LM); (**d**) the deformed area of the face pendant (SEM); (**c**) the rectangular pendant front (SEM); (**f**) the bead made of seven small globules (SEM); (**g**) jewellery fragment (SEM); (**h**) the coil rod at the left side of the jewellery fragment (SEM); and (**i**) the coil at the right side of the jewellery fragment (SEM). Photo copyright holder: D. Ashkenazi and O. Tal.

According to the chemical analysis results, all objects were produced from a similar type of silver alloy containing a small wt% amount of copper (Fig. 4). The average measured copper concentration in the jewellery from the Samaria Hoard was 6.6 ± 1.6 wt% Cu; and the average amount of Cu in the jewellery from the Nablus Hoard was 5.1 ± 1.9 wt% Cu (excluding the joining areas and the unique near-eutectic materials). The average measured copper concentration in the joints (both brazing and CM) from both hoards was 17.0 ± 10.0 wt% Cu (Fig. 4).

**Figure 6:**
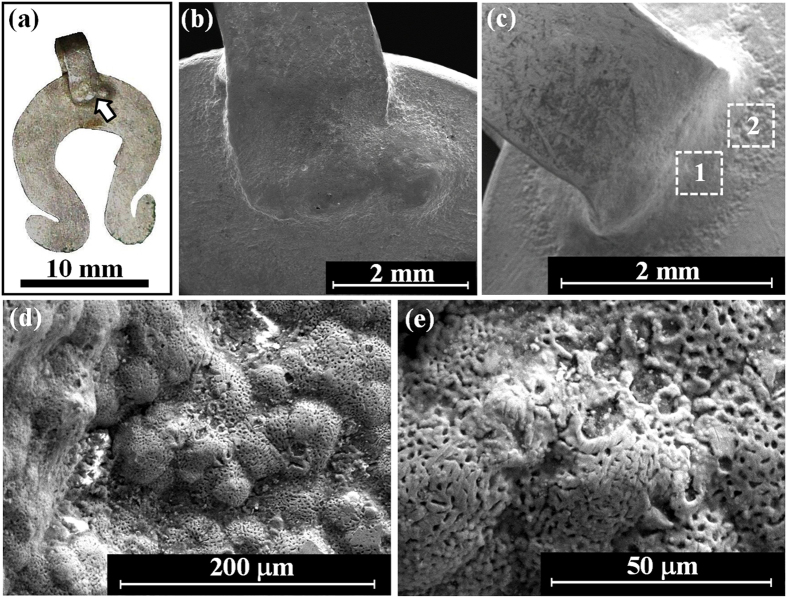
The omega pendant from the Nablus Hoard: (**a**) general view of the examined joint (arrow, front); (**b**) the joint between the loop and the pendant (front, SEM); (**c**) the joint between the loop and the pendant (back, dash squares, SEM); (**d**) the brazed area (back, inside dash square 2, SEM); and (**e**) higher SEM magnification (x1542) of the area inside the dash square 2 showing the morphology of the surface. Photo copyright holder: D. Ashkenazi and O. Tal.

**Figure 7:**
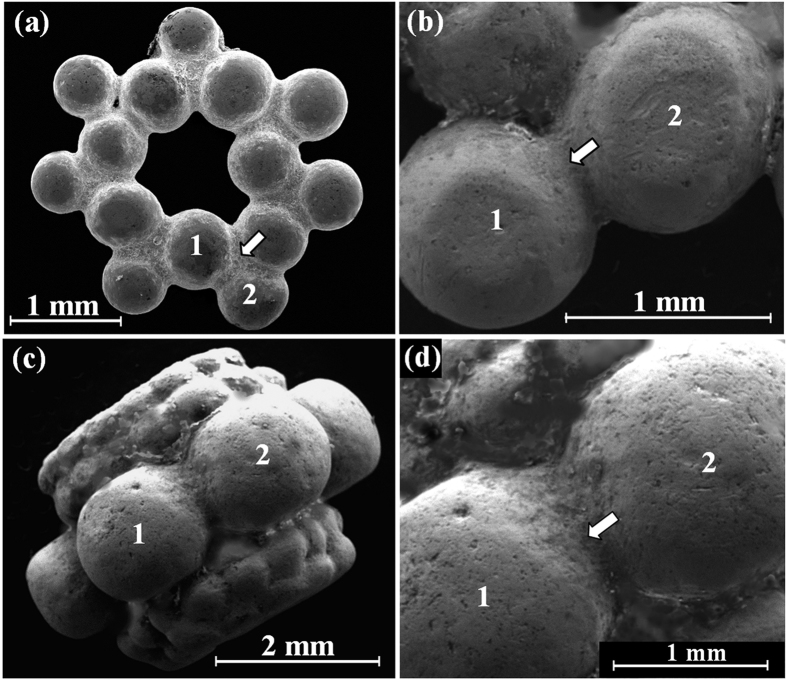
SEM images of the silver beads from the Nablus Hoard, showing: (**a**) bead 1; (**b**) bead 2 examined joint between two globules (arrow); (**c**) bead 3, made of large (1.5–1.8 mm) and small (500 μm) joint globules; and (**d**) bead 3, joint between globules 1 and 2 (arrow). Photo copyright holder: D. Ashkenazi and O. Tal.

**Figure 8:**
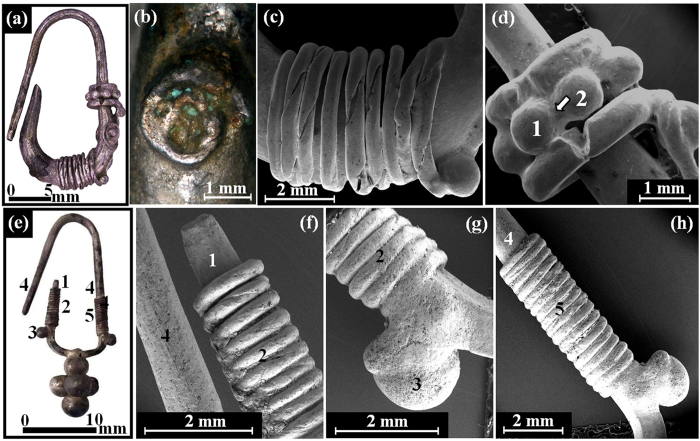
Images of the silver earrings from the Nablus Hoard, showing: (**a**) general view of earring A (side A); (**b**) the area of a missing globule in earring A (side B, stereo LM); (**c**) the coil of earring A and the granule at its end (SEM); (**d**) the bead at the right side of earring A (SEM); (**e**) areas 1–5 of earring B (side A); (**f**) the bar (areas 1 and 4) and the coil (area 2) on the left side of earring B (SEM); (**g**) the coil (area 2) with granule at its end (area 3); and (**h**) the bar and the coil (area 5) with granule at its end, at the right side of earring B. Photo copyright holder: D. Ashkenazi and O. Tal.

Much higher copper concentrations were measured in the joining regions manufactured by both CM and brazing (Tables 1 and 2). The results indicate that in all jewellery pieces from both hoards, copper was used as a joining element between silver parts. Since various joining methods were observed, including **mechanical joints, narrow gap CM** and **wide gap brazing**, a NDT metallurgical methodology was developed (Table 3) based on the composition of the bulk and joints of the jewellery. For example, in the jewellery fragment from the Samarian Hoard (Fig. 2e), a wide gap joint was observed; therefore, based on the developed methodology, a brazing technique was most likely used, and indeed this assumption is supported by the analysis results of the joint (with above eutectic alloy composition of 38.6 wt% Cu). An exceptional example was revealed in the case of the beads from both hoards (Figs 2d and 3f). A narrow gap multi-joint was observed; therefore, based on the developed methodology, it can be said that the granules were joined together simultaneously to form a bead by copper CM. The analysis results revealed a high concentration of Cu at the joints. For instance, the examined composition of the Samarian hoard bead at its joints was 14.4–29.4 wt% Cu, and the composition of bead 1 from the Nablus hoard at its examined joint was 42.4 wt% Cu. These high copper values at the joints of beads may be explained according to presence of copper residues, which remained on the surface of the joints. Another explanation, supported by the developed methodology, may be the use of a two-step manufacturing process: first, the beads were joined by CM, and then, the joint quality was improved by applying an additional CM cycle. In the case of ring B, based on the observed narrow gap single-joint and on the developed methodology, the ring was joined by CM, as supported by the chemical analysis results of the joint (8.8–9.5 wt% Cu). In the omega pendant, based on the observed wide gap joint (Fig. 6a–c) and on the developed methodology, the pendant and its loop were joined by brazing, as supported by the chemical analysis results of the joint (with 9.7 wt% Cu at the front side of the pendant and 20.1–29.7 wt% Cu at the back side of the pendant). Based on VT and since there was a compositional difference between the front and back joints of the omega pendant, the front joint (with higher copper concentrations, Table 2) was probably repaired. In the case of earring A, relatively narrow gap multi-joints were observed (Fig. 8a–d). Therefore, according to the results of the applied methodology, the earring was joined by CM, as supported by the analysis results of the joints (7.5–13.7 wt% Cu). In earring B, again relatively narrow gap multi-joints were observed (Fig. 8e–h). Therefore, according to the applied methodology, the earring was joined by CM, as supported by the analysis results of the joints (8.1–8.9 wt% Cu). Based on VT, the large globules were probably connected by CM; however, they were brazed to the square cross-section of earring B’s rod (arrow, Fig. 8e).

In special circumstances, as in the case of ring A from the Samaria Hoard (with 12.4–25.3 wt% Cu), the leaf-shaped pendant B and bead 1 from the Nablus Hoard (with 13.6–35.7 wt% Cu and 13.2–14.7 wt% respectively), the high amount of copper may indicate that a near eutectic alloy, normally employed in the brazing process, was used to manufacture these objects^41^. These results most likely indicate that near eutectic alloys were produced and used for brazing jewellery parts during their fabrication and the leftovers were occasionally used for artefact production themselves.

There is a similarity between the developed methodology and the chemical analysis results of most of the examined silver objects, including the jewellery fragment from the Samaria Hoard (wide gap joint, brazing, 38.6 wt% Cu); ring B from the Nablus Hoard (narrow gap, CM, 8.8–9.5 wt% Cu), the omega pendant from the Nablus Hoard (9.7 wt% Cu at the repaired front; and wide gap, brazing, 20.1–29.7 wt% Cu at the back); earring A from the Nablus Hoard (narrow gap multi-joints, CM, 7.5–13.7 wt% Cu); and earring B from the Nablus Hoard (narrow gap multi-joints, CM, 8.1–8.9 wt% Cu). There is partial similarity between the developed methodology and the chemical analysis results of the beads from both hoards. In the case of the joints between the globules, based on the chemical analysis results shown in Tables 1 and 2, it is hard to distinguish between CM and brazing. Since all examined globules are tiny in size (for example, the average diameter of the globules from the Nablus Hoard’s beads 1 and 2 is 1.0 mm), it was extremely difficult for the ancient silversmiths to control the joining process of such globules. Therefore, it is concluded that the developed methodology gives good results when the examined joint is between two relatively flat surfaces. However, when the CM joint is between two spheres (two points) and the examined area is curved, as in the case of the joins between the globules, the methodology has limitations and should be improved in the future. Hence, it is recommended in future studies to examine the composition of other beads produced of tiny globules to reveal more information on their joining methods.

The manufacturing practices of the jewellery from both hoards involved parallel techniques, including casting, thin foil manufacturing, granulation and joining, indicating that the objects were prepared by highly trained silversmiths. The silversmiths used a near eutectic sliver-copper brazing alloy and heated the object to a temperature well below the melting point of the silver (Fig. 4). This silver brazing technique was a common method in antiquity that used the lower melting point of Ag-Cu alloys to join silver parts^57^. Another example of sophisticated metalwork technology that was popular during antiquity is the granulation technology^46^,^58^.

Prestige objects such as silver jewellery have a significant historical value and as a result, their metallurgical examination may give valuable information regarding the local material culture of a specific period. Silver was considered one of the most valuable metals in antiquity and was more commonly used than gold. It is frequently part of hoards and treasures found mostly in what is defined as *Hacksilber* (irregularly cut silver), broken pieces of silver ingots and jewellery used as currency or money. Material in this form was weighed on scales against standardized weights for exchange or payment before and after the development of coinages. In the case of the Samaria and Nablus Hoards, the silver items are still defined as jewellery and not *Hacksilber* since they are not broken and were added to the hoard because they were valuable objects. Therefore, the jewellery of both hoards seems to be of high quality. Since metals such as silver-copper alloys are less expensive than pure silver, the presence of high copper content in an artefact may also result from local economic considerations^15^,^59^. Although the Samaria Hoard – 352 BCE – is dated 21 years earlier than the Nablus Hoard – 331 BCE, based on the shape, composition and manufacturing techniques of the artefacts, there is a high level of continuity in the local Samaritan production technology of silver jewellery during the Persian period. Hence, the silversmiths of both hoards probably received their technical knowledge and training at the same workshop; consequently, it is extremely likely that the jewellery of both hoards were manufactured at the same central workshop. This information provides better understanding of Persian-period Samarian technological abilities and material culture including the coins they produced.

A metallurgical methodology has been developed here (Table 3), based on the composition of the joining Ag-Ag regions and bulk regions of the silver jewellery in order to study the jewellery manufacturing processes in local Samarian society and to examine possible continuity between the two hoards during the 351–331 BCE period. This information provides better understanding of the Persian-period Samarian technological abilities and material culture. Moreover, this methodology can be applied in the future to similar metal objects from other hoards and may serve as a useful tool in the interpretation of technological progress, continuity, trade relations and economic considerations of different ancient cultures.

## Methods

The NDT examination of silver jewellery from the two hoards included the following methods:

- VT and stereo microscope observations were carried out to identify and detect the quality of the jewellery as well as their joints at the macroscopic level.
- A digital multi-focal LM was used to determine the quality of the items as well as their joints at the microscopic level. The observations were made with high intensity LED lighting (5700 K colour temperature) and high resolution HD (1920 × 1200), as well as an auto-focus and multi-focus system.
- An environmental examination was performed at the Wolfson Applied Materials Research Centre, Tel Aviv University, using environmental SEM (ESEM, Quanta 200 FEG) at high vacuum mode and an Everhart-Thonley secondary electron detector in order to observe the surface of the jewellery and to relate it to the composition and manufacturing process. The composition was analysed by EDS using a Si(Li) liquid-cooled X-ray detector. The EDS was calibrated with standard samples from the manufacturer and provided measurements with a first approximation error of 1%. The EDS elemental composition (Table 1 and Table 2) was obtained by using the ‘no peaks omitted’ spectrum option combined with ‘all elements analysed (normalised)’ processing option, with iteration number of 2–4. The Ag/Cu ratio of the silver alloys was obtained by omitting the peaks of the elements O, Si, Cl, S, Fe, Al, Ca, and Mg directly from the EDS’s Oxford instrument program (INCA Energy EDS X-ray Microanalysis System). For each artefact several areas (each of between 30 × 30 μm^2^ to 150 × 150 μm^2^) were scanned and analysed to examine the composition homogeneity of the objects and to identify joining materials.

## Additional Information

**How to cite this article**: Ashkenazi, D. *et al*. Metallurgical investigation on fourth century BCE silver jewellery of two hoards from Samaria. *Sci. Rep.* **7**, 40659; doi: 10.1038/srep40659 (2017).

**Publisher's note:** Springer Nature remains neutral with regard to jurisdictional claims in published maps and institutional affiliations.

## Figures and Tables

**Figure 1:**
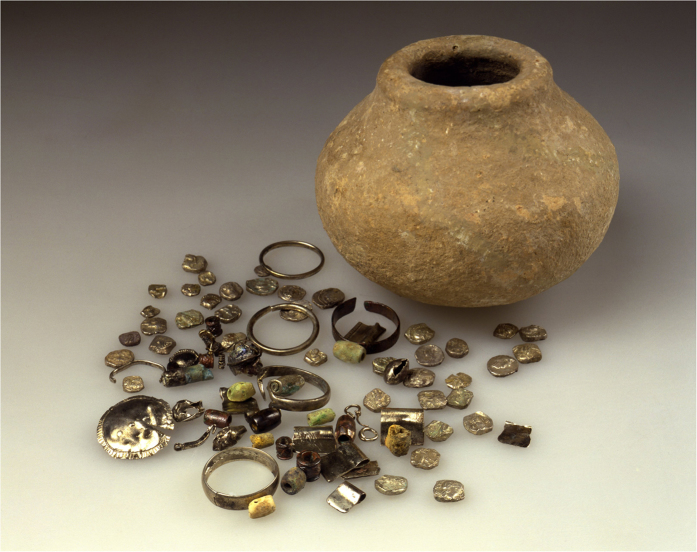
The fourth century BCE Samaria Hoard. The maximum diameter of the vessel is 88 mm and the height of the vessel is 65 mm. Photo with the permission of the Israel Museum. The Israel Museum no. 93.16.14531–14569 Photo ©, Jerusalem. Photographer: D. Harris.

**Table 1:** SEM-EDS analysis results of the silver jewellery from the Samaria Hoard (SA = scanned area).

Sample	Composition (wt %)								
	Surface							Silver alloy	
	Ag	Cu	O	Si	Cl	S	Other	Ag	Cu
Ring A (area 1, Figs 2a and 5a)	63.4	9.0	22.7	2.3	2.6	—	—	87.6	12.4
Ring A (area 2, Figs 2a and 5a,b)	56.2	19.0	16.6	3.1	3.4	0.8	0.9 (Ca)	74.7	25.3
Face pendant (front, Figs 2b and 5c)	79.2	3.8	14.8	0.7	0.8	0.7	—	95.4	4.6
Face pendant (back, Figs 2b and 5d)	74.0	5.9	17.2	0.7	1.6	0.6	—	92.6	7.3
Rectangular pendant (front, area 1, Figs 2c and 5e)	74.2	4.5	16.7	1.4	3.2	—	—	94.3	5.7
Rectangular pendant (front, area 2, Figs 2c and 5e)	76.4	5.0	16.0	1.3	1.3	—	—	93.9	6.1
Bead (globule 1, Figs 2d and 5f)	79.3	3.8	15.3	0.8	0.8	—	—	95.4	4.6
Bead (globule 2, Figs 2d and 5f)	78.0	4.3	11.7	1.1	2.1	1.3	0.5 (Mg), 1.0 (Fe)	94.8	5.2
Bead (joint between globules 1 and 2, Figs 2d and 5f, SA 1: 100 × 100 μm^2^)	54.2	9.1	24.8	4.1	1.5	3.2	0.5 (Mg), 0.8 (Fe), 1.8 (Ca)	85.6	14.4
Bead (joint between globules 1 and 2, Figs 2d and 5f, SA 2: 100 × 100 μm^2^)	60.4	25.2	13.3	—	1.1	—	—	70.6	29.4
Jewellery fragment (area 1, bar, Figs 2e and 5g)	69.9	13.8	13.8	—	2.1	0.4	—	83.5	6.5
Jewellery fragment (area 2, coil/bar joint, Figs 2e and 5g,i, SA: 100 × 100 μm^2^)	48.6	30.6	17.8	0.5	1.2	0.7	0.6 (Fe)	61.4	38.6
Jewellery fragment (area 3, bar, Figs 2e and 5g,j)	77.7	7.5	13.7	0.5	0.6	—	—	91.2	8.8
Jewellery fragment (area 4, coil, Figs 2e and 5g–h)	75.2	8.2	14.9	0.5	0.8	0.4	—	90.2	9.8
Jewellery fragment (area 5, bar, Figs 2e and 5g)	76.9	6.1	9.8	0.9	4.2	0.8	0.7 (Fe), 0.6 (Ca)	92.7	7.3

The approximate error on the EDS measurements of the silver composition (mean value and standard deviation) based on the measurements of 80 silver coins from both the Samaria and Nablus Hoards is 2.5%.

**Table 2:** SEM-EDS analysis results of the silver jewellery from the Nablus Hoard (SA = scanned area).

Sample	Composition (wt %)								
	Surface							Silver alloy	
	Ag	Cu	O	Si	Cl	S	Other	Ag	Cu
Ring B (front, Fig. 3a)	75.2	4.4	18.8	1.0	0.6	—	—	94.6	5.4
Ring B (back)	78.6	4.4	12.6	—	0.9	—	3.5 (Pb)	94.7	5.3
Ring B (back, after grinding)	91.7	3.7	4.0	—	0.6	—	—	96.1	3.9
Ring B (back joint, Fig. 3a, SA: 100 × 100 μm^2^)	74.3	7.2	14.3	—	0.7	—	3.5 (Pb)	91.2	8.8
Ring B (back joint, grinded, SA: 100 × 100 μm^2^)	76.5	8.0	10.0	0.4	1.7	—	3.4 (Pb)	90.5	9.5
Pendant B (front, Fig. 3c)	75.9	12.1	12.0	—	—	—	—	86.4	13.6
Pendant B (front loop, Fig. 3c)	36.4	20.5	35.8	4.5	1.9	—	0.9 (Al)	65.3	35.7
Pendant C (front, Fig. 3d)	94.7	1.8	3.5	—	—	—	—	98.2	1.8
Omega pendant (front, Fig. 3e)	82.1	3.8	14.1	—	—	—	—	95.7	4.3
Omega pendant (front joint, Figs 3e and 6b, SA: 150 × 150 μm^2^)	69.7	7.5	20.2	0.6	—	0.6	1.4 (Fe)	90.3	9.7
Omega pendant (back, Fig. 3e)	73.6	4.9	20.4	—	—	—	1.1 (Fe)	93.8	6.2
Omega pendant (back loop, Fig. 3e)	61.6	4.6	26.4	5.1	—	—	2.3 (Fe)	93.1	6.9
Omega pendant (back joint, Figs 3e and 6c, SA 1: 80 × 80 μm^2^)	41.9	17.8	35.8	0.4	—	1.4	2.7 (Fe)	70.3	29.7
Omega pendant (back joint, Figs 3e and 6c, SA 2: 80 × 80 μm^2^)	46.0	11.6	35.4	0.7	—	0.9	0.5 (Al), 4.9 (Fe)	79.9	20.1
Bead 1 (globule 1, Figs 3f and 7a)	67.6	10.6	20.4	0.7	0.7	—	—	86.8	13.2
Bead 1 (globule 2, Figs 3f and 7a)	71.8	12.7	15.0	—	—	0.5	—	85.3	14.7
Bead 1 (joint between globules, Fig. 7a, SA: 30 × 30 μm^2^)	37.4	28.9	28.7	1.8	1.1	1.5	0.6 (Al)	57.6	42.4
Bead 2 (globule 1, Figs 3f and 7b)	81.0	3.3	14.5	1.2	—	—	—	96.1	3.9
Bead 2 (globule 2, Figs 3f and 7b)	72.9	7.5	19.3	—	—	—	—	90.7	9.3
Bead 2 (joint between globules, Fig. 7b, SA: 80 × 80 μm^2^)	36.7	6.4	53.1	2.1	—	—	1.7 (Al)	85.2	14.8
Bead 3 (globule 1, Figs 3f and 7c,d)	73.5	6.2	20.3	—	—	—	—	92.2	7.8
Bead 3 (globule 2, Figs 3f and 7c,d)	74.6	6.3	18.7	0.4	—	—	—	92.2	7.8
Bead 3 (globules joint, Fig. 7c,d, SA: 80 × 80 μm^2^)	39.0	18.4	41.9	0.7	—	—	—	67.8	32.2
Earring A (bar near globule, Figs 3g and 8c)	90.8	3.5	5.7	—	—	—	—	96.3	3.7
Earring A (bar above bead, Figs 3g and 8d)	85.2	3.4	11.4	—	—	—	—	96.2	3.8
Earring A (coil, Figs 3g and 8c)	93.0	2.1	4.3	—	0.6	—	—	97.8	2.2
Earing A (coil/globule joint, Figs 3g and 8c, SA: 50 × 50 μm^2^)	81.3	12.9	5.2	—	—	0.6	—	86.3	13.7
Earring A (bar/globule joint, Figs 3g and 8c, SA: 50 × 50 μm^2^)	75.5	6.1	17.4	0.5	—	0.5	—	92.5	7.5
Earring A (bead, globule 1, Figs 3g and 8d)	89.2	3.4	6.8	—	0.6	—	—	96.3	3.7
Earring A (bead, globules joint, Fig. 8d, SA: 80 × 80 μm^2^)	66.2	6.7	26.6	—	—	0.5	—	90.8	9.2
Earring B (bar, area 1, Figs 8e and f)	81.7	2.3	11.3	1.4	0.6	2.7	—	95.4	4.6
Earring B (coil, area 2, Figs 8e,f,g)	67.9	4.0	24.4	1.4	0.8	1.5	—	93.8	6.2
Earring B (coil/globule joint, Fig. 8g, SA 2/3: 50 × 50 μm^2^)	61.3	5.4	27.9	3.2	0.6	1.1	0.5 (Al)	91.1	8.9
Earring B (coil/bar joint, Fig. 8e,f, SA 4/5: 50 × 50 μm^2^)	81.2	7.2	11.1	0.5	—	—	—	91.9	8.1
Earring B (coil, area 5, Fig. 8e,h)	71.7	3.4	19.3	2.2	—	3.4	—	95.5	4.5

The approximate error on the EDS measurements of the silver composition (mean value and standard deviation) based on the measurements of 80 silver coins from both the Samaria and Nablus Hoards is 2.5 %.

**Table 3:** Methodology used for estimation of joining method vs. joint design.

Process	Wide Gap Joint	Narrow Gap Joint
Brazing	Most likely	Feasible
Contact Melting (CM)	Feasible	Most Likely
